# MicroRNA induction by copy number gain is associated with poor outcome in squamous cell carcinoma of the lung

**DOI:** 10.1038/s41598-018-33696-1

**Published:** 2018-10-18

**Authors:** Endi Xia, Sotaro Kanematsu, Yusuke Suenaga, Asmaa Elzawahry, Hitomi Kondo, Noriko Otsuka, Yasumitsu Moriya, Toshihiko Iizasa, Mamoru Kato, Ichiro Yoshino, Sana Yokoi

**Affiliations:** 1 Cancer Genome Center, Chiba Cancer Center Research Institute, Chiba, Japan; 2 Department of General Thoracic Surgery, Graduate School of Medicine, Chiba University, Chiba, Japan; 3 Division of Genetic Diagnostics, Chiba Cancer Center, Chiba, Japan; 4 Department of Bioinformatics, National Cancer Center, Tokyo, Japan; 5 Division of Thoracic Diseases, Chiba Cancer Center, Chiba, Japan

## Abstract

Copy number gains in cancer genomes have been shown to induce oncogene expression and promote carcinogenesis; however, their role in regulating oncogenic microRNAs (onco-miRNAs) remains largely unknown. Our aim was to identify onco-miRNAs induced by copy number gains in human squamous cell carcinoma (Sq) of the lung. We performed a genome-wide screen of onco-miRNAs from 245 Sqs using data sets from RNA-sequencing, comparative genomic hybridization, and the corresponding clinical information from The Cancer Genome Atlas. Among 1001 miRNAs expressed in the samples, 231 were correlated with copy number alternations, with only 11 of these being highly expressed in Sq compared to adenocarcinoma and normal tissues. Notably, miR-296-5p, miR-324-3p, and miR-3928-3p expression was significantly associated with poor prognosis. Multivariate analysis using the Cox proportional hazards model showed that miRNA expression and smoking were independent prognostic factors and were associated with poor prognosis. Furthermore, the three onco-miRNAs inhibited *FAM46C* to induce *MYC* expression, promoting proliferation of Sq cells. We found that copy number gains in Sq of the lung induce onco-miRNA expression that is associated with poor prognosis.

## Introduction

Copy number alternations, such as genomic amplifications and deletions, induce oncogene expression and repress tumor suppressor genes^1^. Even low-level copy number gains have been found to contribute to oncogene induction and have been associated with malignant phenotypes. Typical examples of such oncogenes include the *MYC* family genes^2^ and *HER2*^3^. In lung cancer, for instance, we previously reported that *SKP2* was amplified in a homogenous pattern in small cell carcinoma^4^, but in non-small cell lung carcinoma (NSCLC), a lower *SKP2* copy number gain was necessary to influence metastasis^5^. These reports suggest that even low-level copy number gains in certain oncogenes contribute to carcinogenesis in various cancers. However, these studies mainly focused on well-known oncogenes, and the significance of low-level copy number changes in non-coding genomic regions and lesser known oncogenes in carcinogenesis remains elusive.

Micro (mi) RNAs are one type of small endogenous non-coding RNAs that interact with the 3′ untranslated region (UTR) of target mRNA via partial Watson-Click pairing^6^. The interaction promotes mRNA degradation and/or inhibits protein translation. In cancer, miRNAs have been shown to have tumor suppressive or oncogenic functions related to the inhibition of oncogene or tumor suppressor gene expression, respectively^7,8^. Squamous cell carcinoma (Sq) of the lung is the second most serious subtype of lung cancer^9^. MiRNAs have been shown to play a role in Sq pathogenesis. Furthermore, recent omics-based studies have uncovered somatic alternations in Sq patients^10^, however, the contribution of copy number alternation to miRNA expression in Sq has been poorly characterized.

In this study, we performed a genome-wide oncogenic (onco-) miRNA screening on 245 samples of lung Sq using data sets from the Cancer Genome Atlas (TCGA). By analyzing the data sets from RNA sequencing and comparative genomic hybridization studies, we identified three onco-miRNAs, the expression of which correlated with low-level copy number changes. Furthermore, combinations of three onco-miRNAs, miR-296-5p, miR-324-3p, and miR-3928-3p, had a prognostic impact on survival in lung Sq patients, indicating a significant role in Sq pathology.

## Results

### Identification of miRNAs upregulated in response to copy number gains

**Figure 1:**
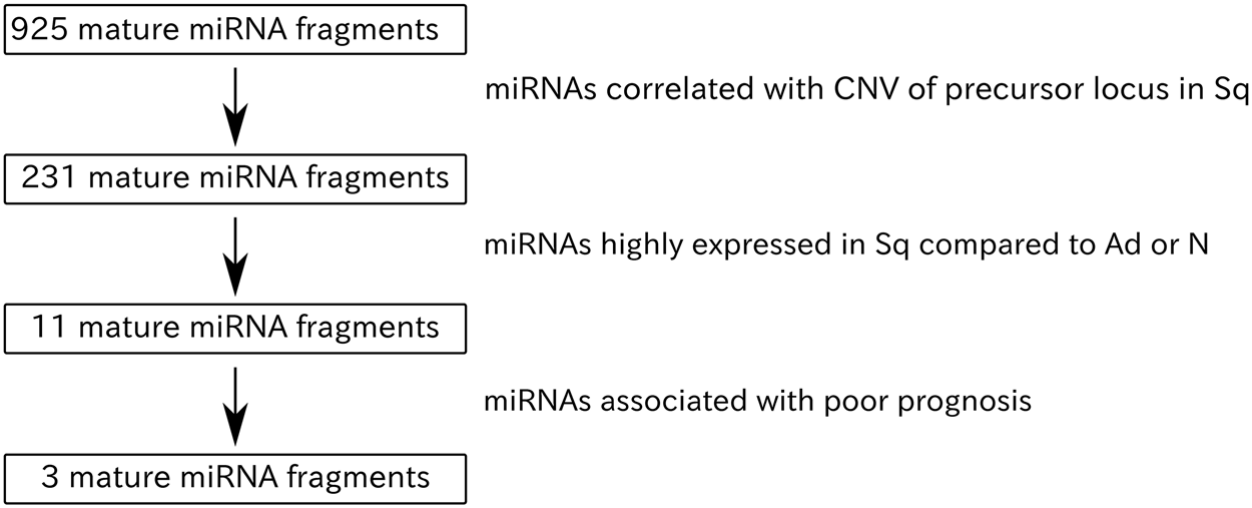
Flow diagram showing the miRNA selection process used in this study.

We analyzed 245 Sq and 239 Ad of the lung as well as 91 normal lung tissue samples, using miRNA sequencing (miRNA-Seq) and copy number alternation data sets from TCGA. The clinical information of all cases investigated is presented in Table 1. In order to investigate the correlation between copy number variation (CNV) and expression level (CPM) at each locus, we calculated the Pearson correlation coefficient (Fig. 1). Out of 925 mature miRNAs (753 precursors), the expression of 231 (processed from 188 loci) was significantly correlated with CNV (Fig. 1; P-value < 0.05; R-value > 0.2). Next, we compared the expression of these 231 mature miRNAs in Sq to that in Ad and normal tissues. Notably, the expression of 11 miRNAs was significantly higher in the Sq samples compared to Ad and normal tissues (Fig. 1; Sq vs Ad: log_2_FC (Fold change) >0, Q < 10^−10^, Sq vs N: log_2_FC > 0, Q < 10^−10^). The 11 mature miRNAs are listed in Supplementary Table S1.

**Table 1:** Clinical characteristics of patients with lung adenocarcinomas (Ad) and squamous cell carcinomas of the lung (Sq). Clinical characteristics of lung cancer patients.

Subjects	Ad (n = 239)	Sq (n = 245)
**Histological subtype**		
**Age**		
Range	40–88	41–90
Mean ± SD	65 ± 10	68 ± 9
**Sex**, **n (%)**		
Male	107 (45)	185 (76)
Female	132 (55)	60 (24)
**Smoking**, **n (%)**		
Smoking	198 (83)	229 (93)
Nonsmoking	33 (14)	10 (4)
Unknown	8 (3)	6 (3)
**Stage**, **n (%)**		
I	134 (56)	119 (48)
II	57 (24)	85 (35)
III	38 (16)	37 (15)
IV	10 (4)	2 (1)
Unknown	0	2 (1)

### High levels of miR-296-5p, miR-324-3p, and miR-3928-3p were correlated with poor prognosis

**Figure 2:**
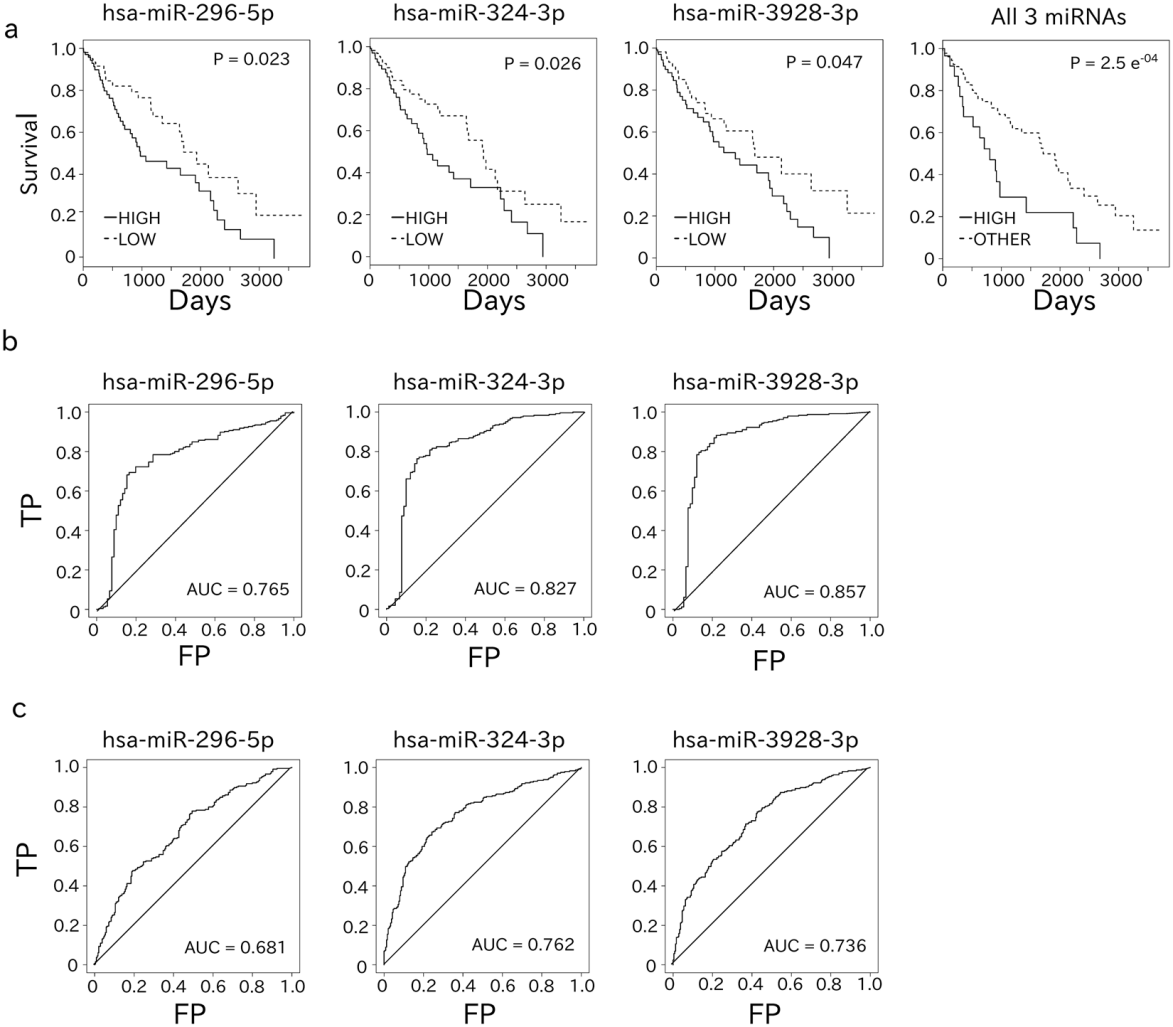
Survival probability and ROC curve for Sq patients according to miRNA expression. (**a**) Kaplan-Meier curve showing that higher miRNA expression was associated with poor prognoses. P-values were calculated by log-rank test. (**b**,**c**) The ROC curve indicates that higher expression of individual miRNAs is a good indicator to separate Sq from normal tissues (N) (**b**) or Sq from Ad (**c**).

**Figure 3:**
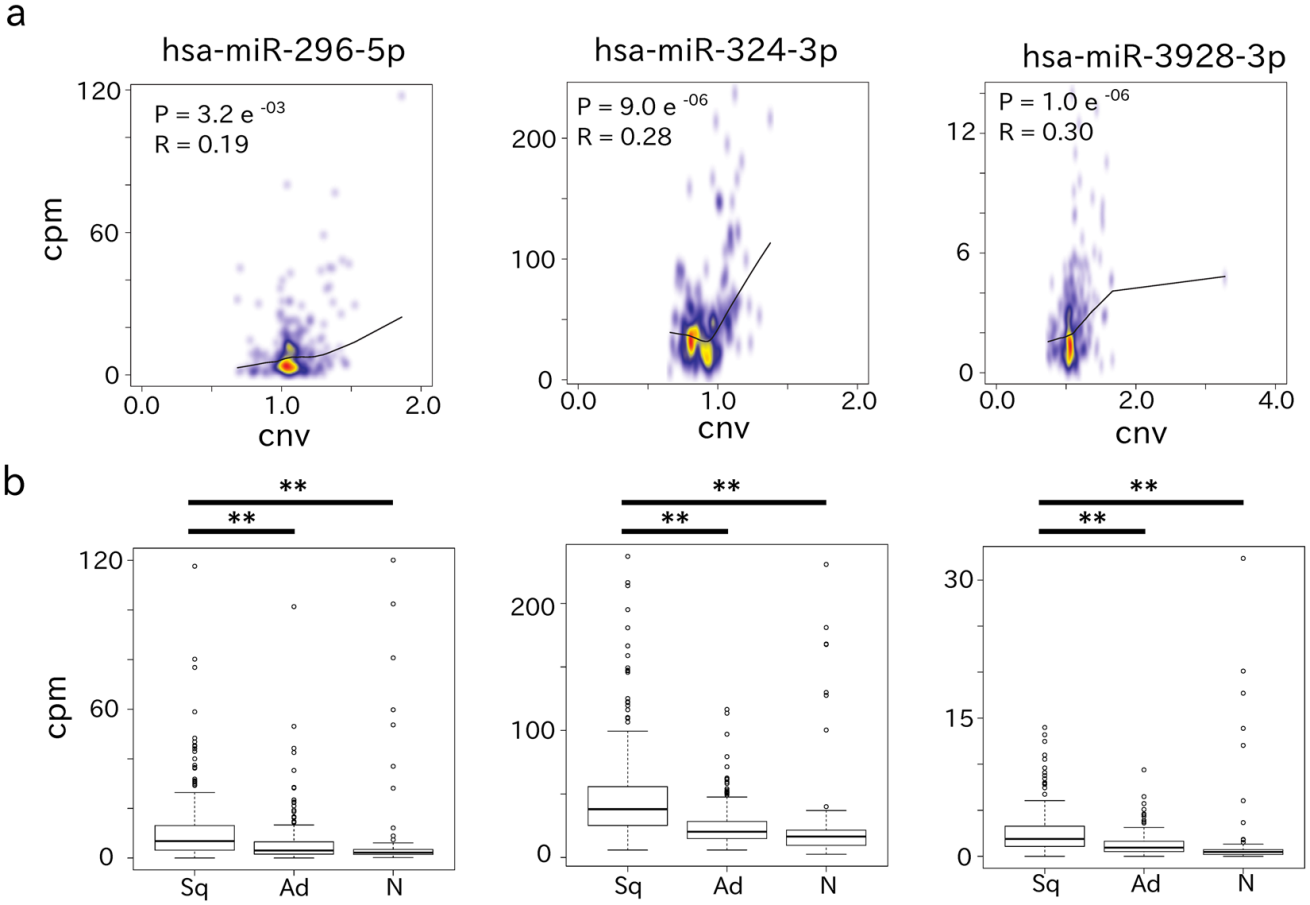
Characteristics of the three miRNAs associated with poor prognoses. (**a**) Density scatter plots showing the association between miRNA (miR-296-5p, miR-324-3p, and miR-3928-3p) expression and CNV in the precursor locus, particularly for copy number gains. The curved line was estimated with R function “lowess”. (**b**) Boxplots showing the higher expression of the three miRNAs of interest in Sq samples relative to those in Ad and normal (N) samples. P-values were calculated with the Wilcoxon rank sum test: (*) P-value < 0.05; (**) P-value < 0.01.

We next examined the prognostic impact of the 11 miRNAs differentially upregulated in Sq to identify onco-miRNAs for this particular cancer. Notably, as five of the Sq data sets did not include the corresponding clinical information, survival rates were not calculated for these patients. For the remaining 240 Sq data sets, patients/samples were divided into high or low expression groups based on being higher or lower than the average value of each miRNA. The log-rank test was then used to evaluate the differences in survival rate between the two groups. Upregulated expression of three miRNAs (miR-296-5p, miR-324-3p, and miR-3928-3p) was significantly correlated with a poor prognosis (Fig. 2a). The prognostic impact of varying combinations of these three miRNAs was then evaluated. This analysis indicated that the combination of miR-296-5p, miR-324-3p, and miR-3928-3p expression was related to the worst clinical prognosis according to the log-rank test (Fig. 2a). The distribution of P-values calculated from a bootstrap re-sampling approach showed that the P-value of the combination of the three miRNAs was much more reproducible than that of each of miR-296-5p, miR-324-3p, and miR-3928-3p (Supplementary Fig. S1). Moreover, the P-value of each of the three miRNAs was much more reproducible than that of each of null model miRNA (miR-15b-3p, miR-942-5p, and miR-27a-3p), which were randomly selected from among the other miRNAs for comparison (Supplementary Fig. S1). Next, we assessed the prognostic impact of the three miRNAs alone and with clinical covariates (age, gender, stage, and smoking) using the Cox proportional hazards model after confirming that the hazard ratios of each variable were time-independent. The analysis indicated that high expression levels of each of the three miRNAs were significantly associated with poor prognosis in both univariate and multivariate models (P-value < 0.05; Table 2). The combination of the three miRNAs was the most significant (the smallest P-value), and smoking was also significant in this model (P-value < 0.05; Table 2). Furthermore, miR-3928-3p expression exhibited the highest area under the receiver operator curve (AUC) compared to normal tissue (Fig. 2b), whereas miR-324-3p showed the second and third highest AUC compared to Ad and normal tissues, respectively (Fig. 2c, Supplementary Table S2). As shown in Fig. 3a, miRNA expression was positively correlated with CNV of the precursor locus for each of the three miRNAs of interest. The expression of these miRNAs in Sq, Ad, and normal tissues (Fig. 3b) further highlighted their upregulation in Sq. Similar expression patterns were confirmed using an miRNA microarray data set (GSE74190) and our own clinical specimens (Supplementary Fig. S2a,b).

**Table 2:** Univariate and multivariate analyses using Cox proportional hazards models.

Model	Variables	Hazard_ratio (lower 95% CI–upper 95% CI)	P-value
**Uivariate**			
miR-296	miR-296	1.75 (1.05–2.90)	*3*.*21E-02*
miR-324	miR-324	1.76 (1.06–2.92)	*2*.*82E-02*
miR-3928	miR-3928	1.67 (1.00–2.79)	*4*.*92E-02*
3 miRNAs high	3 miRNAs high	2.59 (1.54–4.35)	*3*.*30E-04*
Stage	Stage	0.68 (0.37–1.23)	1.96E-01
Smoking	Smoking	1.56 (0.91–2.67)	1.01E-01
Age	Age	0.93 (0.55–1.57)	7.78E-01
Sex	Sex	1.09 (0.59–2.03)	7.77E-01
**Multivariate**			
**Model**	**Variables**	**Hazard_ratio**	**P-value**
miR-296 + clinical covariates	miR-296	1.97 (1.16–3.33)	*1*.*22E-02*
	Stage	0.59 (0.32–1.09)	9.23E-02
	Smoking	1.67 (0.97–2.86)	6.31E-02
	Age	0.90 (0.53–1.53)	7.00E-01
	Sex	1.11 (0.59–2.09)	7.40E-01
miR-324 + clinical covariates	miR-324	1.89 (0.31–0.89)	*1*.*58E-02*
	Stage	0.67 (0.36–1.22)	1.88E-01
	Smoking	1.58 (0.92–2.74)	9.97E-02
	Age	0.885 (0.52–1.52)	6.75E-01
	Sex	1.10 (0.59–2.07)	7.66E-01
miR-3928 + clinical covariates	miR-3928	1.95 (1.14–3.31)	*1*.*41E-02*
	Stage	0.60 (0.32–1.10)	9.57E-02
	Smoking	1.60 (0.93–2.76)	9.27E-02
	Age	0.86 (0.50–1.48)	5.86E-01
	Sex	1.06 (0.56–2.00)	8.53E-01
3 miRNAs high + clinical covariates	3 miRNAs high	2.84 (1.65–4.87)	*1*.*60E-04*
	Stage	0.65 (0.36–1.19)	1.65E-01
	Smoking	1.73 (1.00–2.99)	*4*.*995E-02*
	Age	1.07 (0.61–1.87)	8.05E-01
	Sex	1.12 (0.59–2.10)	7.33E-01
clinical covariates: Stage + Smoking + Age + Sex			
Italic number: P-value < 0.05			
CI: confidential interval			

Univariate and Multivariate analysis Univariate.

### MiR-296-5p, miR-324-3p, and miR-3928-3p inhibited *FAM46C* to promote cell proliferation of Sq-inducing *MYC* expression

**Figure 4:**
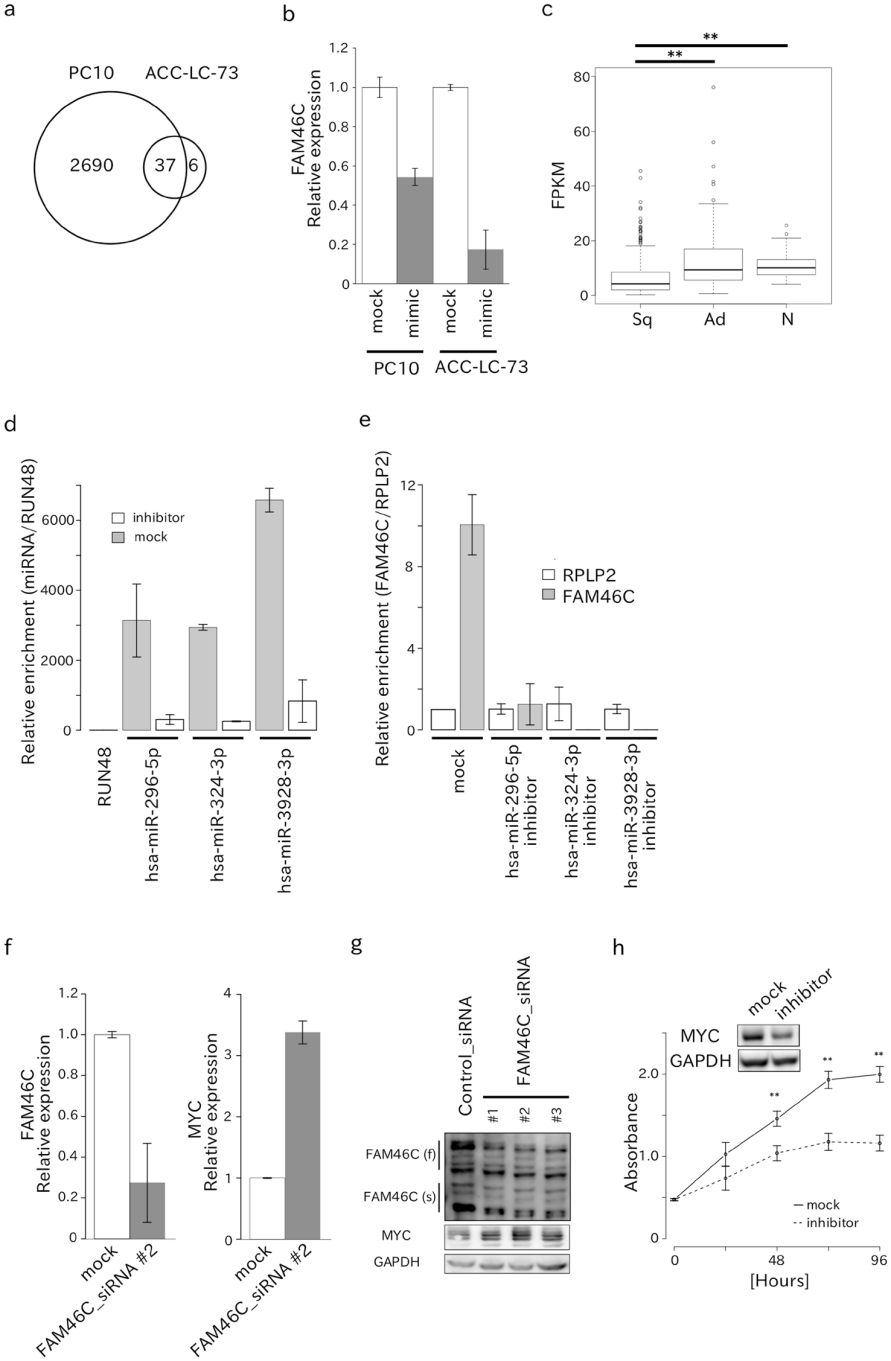
Target prediction and experimental validation. (**a**) Comparison of the common genes downregulated by miR-296-5p, miR-324-3p, and miR-3928-3p in PC10 or ACC-LC-73 cells. (**b**) Barplot demonstrating the ability of miRNA mimics to reduce *FAM46C* mRNA expression in PC10 or ACC-LC-73 cells. (**c**) Boxplot showing the decrease in *FAM46C* expression in Sq compared to Ad and normal tissues (N). P-values were calculated with the Wilcoxon rank sum test: (**) P-value < 0.01. (**d**) Barplot showing the ability of each miRNA to bind to AGO2 protein. (**e**) Barplot showing the ability of *FAM46C* mRNA to bind to AGO2 protein via miRNA. (**f**) Barplot demonstrating the ability of *FAM46C* siRNA to inhibit *FAM46C* mRNA expression in PC10 cells (*left*). Barplot demonstrating the ability of *FAM46C* siRNA to enhance *MYC* mRNA expression in PC10 cells (*right*). Error bars show standard deviations. (**g**) Western blot showing the decrease in FAM46C protein (f: full-length protein, s: short-length protein, ref.^11^) expression and the increase in MYC protein expression upon transfection with *FAM46C* siRNA. (**h**) Polygonal line demonstrating the ability of the combination of the three miRNA inhibitors to suppress the proliferation of PC10 cells. Western blot showing the decrease in MYC protein expression upon transfection of the combination of the three miRNA inhibitors miRNAs in PC10 cells (see also Supplementary Fig. S8).

Next, to determine the mRNA targets of the three miRNAs in lung cancer cells, we performed a microarray analysis. Among a total of 56,689 transcripts, the expression of 2,727 mRNAs were downregulated in the PC10 cells following transfection with onco-miRNA mimics for these three miRNAs (log_2_FC < 0.5). In the ACC-LC-73 cells, the expression of 43 mRNAs were downregulated after onco-miRNA mimic transfection. Only 37 target mRNAs were downregulated in both PC10 and ACC-LC-73 cells (Fig. 4a, Supplementary Table S3). Functional annotation analysis of 37 target mRNAs indicated that these mRNAs were related to apoptosis signaling, cytokine signaling, or IL22 soluble receptor signaling pathways (Supplementary Table S4). Moreover, we found that the 3′ UTRs of three target mRNAs (*FAM46C*, *PPAP2B*, and *WBSCR27*) contained a putative target sequence for the three onco-miRNAs (Supplementary Table S5). Indeed, miRNA mimic transfection decreased *FAM46C* mRNA expression (Fig. 4b). Furthermore, *FAM46C* expression was significantly lower in Sq compared with Ad or normal tissues in the TCGA RNA-Seq data set (Fig. 4c) and was negatively correlated with the three onco-miRNAs (P-value < 0.05; Supplementary Table S6). Next, we performed an RNA immunoprecipitation (RIP) assay using an Argnonaute 2 (*AGO2*) antibody to examine whether the three miRNAs directly bind to *FAM46C* and suppress *FAM46C* expression. We found that the three miRNAs and *FAM46C* were significantly enriched in the IP fraction compared to *RUN48* and *RPLP2*, respectively, and each miRNA inhibitor suppressed the recruitment of *FAM46C* to the *AGO2* complex (Fig. 4d,e). These results indicate that *FAM46C* is regulated by these three miRNAs.

To identify oncogenic pathways related to the three miRNAs, we performed functional annotation analysis using DAVID. The mRNA expression levels of 506 genes were positively correlated with the expression levels of the three miRNAs (P-value < 0.05; Supplementary Table S7), and genes related to cell division, cell cycle, or mitosis were significantly enriched in the gene set (P-value < 0.05; Supplementary Table S8). Furthermore, 29 transcription factors were predicted to be upstream regulators of the 506 genes (Supplementary Table S9). Recently, Zhu *et al*. reported that *FAM46C* downregulates *MYC* expression in myeloma cells^11^. The 29 transcription factors include *MYC* pathway, such as *MYCMAX* and *NMYC*. We thus validated the downregulation of *MYC* expression by *FAM46C* in Sq cells. SiRNA-mediated knockdown of *FAM46C* induced both *MYC* mRNA expression and MYC protein expression in PC10 cells (Fig. 4f,g). Moreover, we introduced miRNA inhibitors or mimics into Sq cell lines. Then, miRNA inhibitors downregulated MYC protein expression and suppressed the proliferation of PC10 cells (Fig. 4h), whereas miRNA mimics upregulated MYC protein expression, and promoted cell proliferation in ACC-LC-73 cells (Supplementary Fig. S3). This suppression of cell proliferation was also observed in multiple miRNA-expressing cell lines (Supplementary Fig. S4a,b), although cell proliferation was not promoted by transfection with miRNA mimics in any of cell lines except ACC-LC-73. Under our experimental conditions, the three miRNAs did not affect cell migration or invasion of PC10 cells (Supplementary Fig. S5). Next, to evaluate the relationship between FAM46C and MYC protein expression levels after transfection with the combined miRNA inhibitors/mimics, we examined the protein levels at 1, 3, 6, 12, 24, 36 h after transfection. Interestingly, the up-regulation of FAM46C protein expression in PC10 started at 1 h after miRNA inhibitor transfection, reached the peak at 3 h after the transfection and fallen, whereas the MYC protein expression level was time-dependently decreased after miRNA inhibitor transfection (Supplementary Fig. S6a). The FAM46C protein expression level in ACC-LC-73 was time-dependently decreased after miRNA mimic transfection, whereas, the MYC protein expression level was time-dependently increased after miRNA mimic transfection (Supplementary Fig. S6b). The similar pattern of the FAM46C expression change was observed in HS24 and PC1 cells (Supplementary Fig. S7a,b). Finally, we evaluated cell growth independently by transfecting each miRNA inhibitor into PC10 to examine the effect of each miRNA on cell proliferation. We found that miR-3928-3p inhibitor most effectively suppressed cell proliferation, increased FAM46C protein expression, and the effect of each miRNA on MYC protein expression was negligible compared to the simultaneous inhibition of the three miRNAs (Supplementary Figs S6, S8a,b). We identified other target genes whose expression was regulated by the three miRNAs (Supplementary S8c), and confirmed that *MYC* was not the direct target gene of the three miRNAs (Supplementary S8d).

## Discussion

Cancer pathogenesis has been linked to both copy number alterations and miRNA expression changes; however, the link between these two cancer-related modifications is largely unknown. In this study, we identified three onco-miRNAs, the expression of which is correlated with copy number alternations, in Sq of the lung. The genomic loci of the onco-miRNAs showed low-level copy number gains. Similar to cancers with low-levels copy number gains for well-known oncogenes, the number of cells with copy number gains can vary in each carcinoma, thus each Sq will have its own distinct levels of onco-miRNAs.In this study, we screened for upregulated Sq-specific miRNAs. A total of 11 miRNAs were highly expressed in Sq compared to Ad or normal tissues, and the high expression of these miRNAs was related to low-level copy number gains. Independent miRNA expression data also showed that expression of miR-296-5p and miR-324-3p was high in Sq. A specific three-miRNA combination involving miR-296-5p, miR-324-3p, and miR-3928-3p showed the strongest prognostic impact on overall patient survival in both univariate and multivariate analyses. It is important to note that the bootstrap analysis showed that the P-value of the three-miRNA combination was reproducible and was not affected by sampling bias. Multivariate analysis showed that smoking history was independently associated with poor prognosis. This result seems reasonable, as smoking is a strong risk factor for Sq^12^. Meanwhile, these three miRNAs exhibited the highest sensitivity, specificity, and AUC values among all of the upregulated Sq-specific miRNAs (Supplementary Table S4). Thus, they could be used not only as prognostic factors, but also as diagnostic factors.

Our analyses revealed that the three miRNAs inhibit *FAM46C* to promote cell proliferation of Sq by binding to the 3′ UTR of *FAM46C*. The *FAM46C* gene is located at the 1p12 locus, which is frequently deleted and significantly mutated in myeloma^13,14^, although we did not find significant mutations in Sq samples (data not shown). *FAM46C* encodes a eukaryotic non-canonical poly (A) polymerase^15^. Silencing of *FAM46C* expression in primary gastric cancer was found to be a biomarker for metastasis or recurrence^16^, and gene knockdown in hepatocellular carcinoma suppressed the anti-metastatic effects of norcantharidin^17^. In our analysis, *FAM46C* mRNA expression was not significantly associated with prognosis in the TCGA data overall, even though a multitude of individual data sets showed a significant association between *FAM46C* expression and favorable overall survival in Sq (data not shown; GSE4573, P-value < 0.05). Loss of *FAM46C* in myeloma promotes cell survival inducing *MYC* expression. Consistent with the role of *FAM46C* in *MYC* regulation, our results showed that knockdown of *FAM46C* induced *MYC* expression in Sq cells. In addition, we found that each miRNA inhibitor induced *FAM46C* expression, showing a negligible effect on *MYC* expression, whereas the simultaneous transfection of the three miRNA inhibitors strongly suppressed *MYC* expression in PC10 cells. Moreover, of the 2727 genes whose expression was downregulated by miRNA mimics transfection, 165 genes were predicted as target genes of the three miRNAs. Functional annotation analysis revealed that the genes related to the Wnt signaling pathway were enriched in the gene set (Supplementary Table S10), and that Wnt-β-catenin inhibitors such as *NKD1*^18^, *HIC1*^19^, *PAX7*^20^, and *TRABD2B*^21^ are direct target genes of the three miRNAs (Supplementary Fig. 3c). Taking into account that *MYC* is a direct target of β-catenin, these results suggest that the three miRNAs may compensate for each other to maintain *MYC* expression in Sq cells and that their simultaneous inhibition is required for abrogation of the oncogenic network between the three onco-miRNAs and the *MYC* pathway.

It is important to note that this is not the first study to recognize the importance of miR-324 and miR-296 in lung cancer. Recently, the prognostic value of miR-324-3p in the early stages Sq of the lung was reported, as demonstrated by the high expression of this miRNA in the plasma of stage I Sq patients compared to health controls and other lung cancers^22^. In the present study, we found that miR-324-3p expression in Sq tissues was high compared to Ad and normal tissues and was correlated with poor prognosis. Therefore, unlike the previous study that focused on the prognostic importance of miR-324-3p expression in early Sq stages, our data suggest that this miRNA could be used as a prognostic marker of Sq of the lung in later stages as well and is not exclusive to the early stages.

Furthermore, miR-296 was also previously found to be upregulated in NSCLCs^23^ and associated with poor prognosis in oral^24^, laryngeal^25^, and esophageal Sq^26,27^ as well as anaplastic gliomas^28^. MiR-296 is hypothesized to promote tumor progression^27^, metastasis, and/or recurrence^25^ by inducing angiogenesis^29^ and invasion^30^ in Sq and gliomas, while it appears to have tumor suppressor functions in other cancers^31,32^. The functional discrepancies observed for this miRNA may be attributed to the varying expression of target genes as well as regulatory genes in different cancer types.

To the best of our knowledge, the present study is the first report showing a significant correlation between lung cancer prognosis and miR-3928-3p. MiR-3928 directly suppresses Dicer^33^, the miRNA biogenesis gene frequently downregulated in NSCLCs^34^. Silencing of Dicer has been shown to promote cancer aggressiveness^7,35^, at least in part, via promotion of metastasis^36,37^. In our results, miR-3928-3p expression more accurately discriminates Sq from normal tissues compared to miR-324-3p expression^17^. Further study is required to examine the significance of onco-miRNA expression in the plasma and how they can be used to predict the risk for Sq of the lung. Targeting these onco-miRNAs as a therapeutic option for Sq patients should also be explored.

In conclusion, we identified three onco-miRNAs that are correlated with low-level copy number gains in Sq of the lung using a genome-wide screen of 245 data sets from TCGA. Furthermore, our results also indicate the prognostic and diagnostic value of these factors. The three onco-miRNAs inhibited *FAM46C* to induce *MYC* expression, promoting proliferation of Sq cells. This study provides insight into the mechanisms underlying Sq pathogenesis in the lung while also providing a foundation upon which additional functional characterizations of these onco-miRNAs can be based. As their functions become clearer, the application of these onco-miRNAs in diagnosing Sq and in the discovery of novel drug targets will likely play a pivotal role in enhancing treatment of aggressive Sq.

## Materials and Methods

### Data collection

All TCGA data were obtained via the TCGA data portal (https://gdc-portal.nci.nih.gov/). Briefly, we collected 245, 239, and 91 expression data sets (RNA-Seq and miRNA-Seq) corresponding to Sq, adenocarcinomas (Ad), and normal tissues, respectively. To evaluate the associations between miRNA expression and CNV in the miRNA precursor loci, we used Sq segment-mean value data sets. The clinical data sets for 245 Sq patients were also collected. To evaluate the reproducibility of miRNA expression patterns in Sq, Ad, and normal tissues, we obtained a data set (GSE74190) via the Gene Expression Omnibus (https://www.ncbi.nlm.nih.gov/geo/).

### Patients

Human tissue specimens were surgically resected from 36 lung Sq patients at the Chiba Cancer Center. The study was conducted in compliance with ethical guidelines for clinical studies in Japan and approved by the Review Board of the Chiba Cancer Center. All patients provided written informed consent before registration. Tumor samples were stored at −80 °C until use.

### Pearson correlation coefficient analysis

Segment-mean values in distinct genomic loci were extracted from TCGA SNP array data (*hg19.seg.txt file). We evaluated CNV using the segment-mean value (log_2_ (CN/2)) as the copy number ratio. We assigned the locus of each miRNA precursor to the copy number ratio using the Bioconductor package “CNTools”. For calculating miRNA expression, we excluded fragments defined as precursor, stemloop, or unannotated. We next obtained the genomic coordinates for mature and precursor miRNAs via Mirbase version 19 (http://www.mirbase.org/) and excluded miRNAs located on the X and Y chromosomes. Expression of a mature miRNA is the sum of the expression of multiple isoforms located in distinct genomic loci. To explore the effects of CNV on gene expression, we calculated the Pearson correlation coefficient between miRNA expression and CNV. MiRNAs with R-value > 0.2 and P-value < 0.05 were considered correlated to CNV. Scatter-plots were prepared using the R functions “smoothscatter” and “lowess”.

### Differentially expressed miRNA (DEMI) analysis

We extracted the digital tag counts of individual miRNA fragment from TCGA miRNA-Seq data (*isoform.quantification.txt file) to calculate the read counts of each miRNA isoform. After excluding dead, disputed, and chromosome X or Y miRNAs using Mirbase (version 19) (http://www.mirbase.org/), we summed the read counts for each miRNA fragment, 1001 of which were mature miRNA fragments with specific MIMAT-IDs. The read counts for each miRNA were then normalized to the total number of mapped read counts per million (CPM). We then performed a DEMI analysis on the 231 miRNAs that were correlated to CNV in the Pearson correlation coefficient analysis to detect differentially expressed miRNAs (DEMI) in Sq relative to Ad or normal controls. MiRNAs with q-value (P-value adjusted by false discovery rate) <10^−10^ were considered differentially expressed.

### Survival analysis

To determine which miRNAs are related to poor prognosis, we set the median expression of each miRNA as the cut-off value, classified the 245 Sq samples into high or low expression groups using this threshold, and compared survival rates between the two groups. Survival curves were estimated using the Kaplan–Meier method. We performed the log-rank test using the R function “survdiff” in the “survival” library, and miRNAs associated with prognosis were defined as those with P-values < 0.05, which corresponded to q-values < 0.2. To check the stability of the calculated P-values, we used a bootstrapping approach, where we randomly re-sampled patient samples with replacement 10,000 times and calculated P-values using the log-rank test for all 10,000 replications. We counted the number of replications that had P-values < 0.05.

We used the R function “coxph” for the Cox proportional hazards model and used the R function “cox.zph” to test the proportional hazards assumption for the Cox model. Clinical factors were categorized, with age classified as high or low based on the median. For smoking classification, the patient was considered a smoker if smoking status was “current smoker”; otherwise the patient was considered a non-smoker. Stage was considered high for stage III or stage IV; otherwise, stage was considered low.

### Receiver operating characteristic (ROC) analysis

To determine whether we were able to distinguish between Sq and Ad or between Sq and normal tissues based on the expression levels of the three miRNAs, we calculated the AUC using R package “ROCR”.

### Target prediction

We used the TargetScan (version 7.1) database (http://www.targetscan.org) to predict the target genes of three miRNAs of interest: miR-296-5p, miR-324-3p, and miR-3928-3p. Briefly, we obtained all target gene lists from the TargetScan database (Summary.count.txt) and extracted the target genes of each miRNA of interest.

### Functional annotation analysis

The Database for Annotation, Visualization and Integrated Discovery (DAVID) (https://www.david.ncifcrf.gov) was used to identify enriched molecular functions and pathways.

### Cell culture

The human squamous lung cancer cell lines ACC-LC-73, HS24, PC1, PC10, and SK-MES-1 were cultured in Roswell Park Memorial Institute medium (RPMI1640, Sigma, Tokyo, Japan) supplemented with 10% fetal bovine serum (FBS) at 37 °C in a humidified atmosphere with 5% CO_2_.

### MiRNA, or *FAM46C* siRNA transfection and RT-qPCR

ACC-LC-73, HS24, PC1, PC10, and SK-MES-1 cells were grown to 40–60% confluency in 12-well cell culture plates (Falcon, Tokyo, Japan), and then the medium was changed to Opti-MEM (Thermo Fisher Scientific, Tokyo, Japan). A total of 40 pmol of miRNA mimic/inhibitor (Thermo Fisher Scientific, Tokyo, Japan), miRNA inhibitor (Thermo Fisher Scientific, Tokyo, Japan), or *FAM46C* siRNA (Sigma, Tokyo, Japan) was transfected into cells using Lipofectamine 2000 (Thermo Fisher Scientific, Tokyo, Japan) according to the manufacturer’s instructions. After incubating for 24 hours, the cell medium was changed to RPMI1640 supplemented with 10% FBS. Transfected cells were collected at 48 hours after transfection. Total RNA was isolated from cells using TRIzol reagent (Thermo Fisher Scientific, Tokyo, Japan), and 1 μg was reverse-transcribed into cDNA using ReverTra Ace (TOYOBO, Osaka, Japan). Expression of *FAM46C* or *GAPDH* mRNA was examined using Step One (Thermo Fisher Scientific, Rockford, IL, USA) and the TaqMan gene expression assay (*FAM46C*: hs00214530, *GAPDH*: 4326317E). Expression of *MYC* mRNA was examined using Step One and KOD SYBR qPCR Mix (TOYOBO, Osaka, Japan) with the following primer sequences: 5′-GGTCTCCACACATCAGCACAA-3′, 5′-TCTTGGCAGCAGGATAGTCCTT-3′. Relative expression was evaluated with the ∆∆Ct method. All RT-qPCRs were performed in triplicate.

### RNA immunoprecipitation (RIP)

RIP assay was performed as described previously^38^. PC10 cells (1 × 10^7^) were washed twice with ice-cold PBS. PC10 cells were gently lysed in 1 mL lysis buffer (20 mM HEPES-NaOH [pH 7.5], 150 mM NaCl, 50 mM NaF, 10 mM Na_3_VO_4_, 1% digitonin, protease inhibitor cocktail [Roche, Basel, Switzerland], 100 U/mL RNasin [Promega, Madison, WI, USA]). The cell lysate was cleared by centrifugation at 15,000 × *g* for 10 min at 4 °C. Immunoprecipitation (IP) was performed for 3 h at 4 °C using anti-*AGO2* specific monoclonal antibodies (Wako, Osaka, Japan) coupled to protein-G beads (Dynabeads, Invitrogen, Carlsbad, CA, USA). The immunoprecipitate was washed three times with wash buffer (50 mM HEPES-NaOH [pH 7.5] 150 mM NaCl, 0.1% Triton X-100). MiRNAs and mRNAs bound to AGO2 protein were extracted using Trizol (Thermo Fisher Scientific, IL WalthamMA, USA). The enrichment of the mRNAs and miRNAs was validated by quantitative real-time RT-PCR analysis (qRT-PCR). Primer sequences are listed in Supplementary Table S11. The fold-enrichment of the signals was detected by the delta-delta Ct method.

### Western blotting

We resolved cell proteins by Sodium Dodecyl Sulfate-PolyAcrylamide Gel Electrophoresis prior to electroblotting onto a Polyvinylidene Difluoride membrane. We incubated the membranes with the following primary antibodies for 1 hour at approximately 25 °C: anti-FAM46C (1:1000 dilution; Abcam Cambridge, UK), anti-MYC (1:1000 dilution; Cell Signaling Technology, Danvers, MA, USA), anti-GAPDH (1:1000 dilution; Thermo Fisher Scientific, Rockford, IL, USA). The membranes were then incubated with a horseradish peroxidase-conjugated secondary antibody (anti-rabbit IgG at a 1:5000 dilution or anti-mouse IgG at a 1:5000 dilution; Cell Signaling Technology), and the bound proteins were visualized using a chemiluminescence-based detection kit (ImmunoStar Zeta or LD; Wako, Osaka, Japan).

### 3-(4,5-dimethylthiazol-2-yl)-2,5-diphenyltetrazolium bromide (MTT) assay

10 µL of water-soluble MTT dye was directly added to the transfected cells in a 96-well plate. After 2 hours of incubation, absorbances at 450 nm were measured using an MTP-310 system plate reader (CORONA, Tokyo, Japan).

### Cell invasion and migration assays

Cell invasion and migration assays were performed using the CytoSelect^TM^ Cell invasion and migration kit according to the manufacturer’s instructions. Transfected cells were cultured in serum-free media for 24 hours, and invasive or migratory cells were lysed and quantified using CyQuant GR Fluorescent Dye. Fluorescence was detected using an ARVO^TM^ X3 system (Perkin Elmer, Tokyo, Japan).

### Microarray analysis

We used a SurePrint G3 GEx Human V3 expression microarray (Agilent Technologies, Tokyo, Japan). A total of 200 ng of RNA was amplified and labeled with cyanine 3 (Cy-3). For hybridization, 600 ng of Cy-3 cRNA was hybridized to an Agilent SurePrint G3 Gex Human V3 microarray. Microarrays were scanned, and the intensity values were quantified using a Surescan Microarray Scanner (Agilent Technologies, Tokyo, Japan). After background subtractions, the raw intensity data was analyzed using the Bioconductor package “Agilp”. We uploaded the microarray data to the Gene Expression Omnibus (GSE115854).

### Statistical analyses

All statistical analyses were performed with R (http://www.r-project.org/). Q-values (P-values adjusted by false discovery rate) were calculated with the Benjamini-Hochberg method.

## Electronic supplementary material

Supplementary figures

Supplementary Table S1

Supplementary Table S2

Supplementary Table S3

Supplementary Table S4

Supplementary Table S5

Supplementary Table S6

Supplementary Table S7

Supplementary Table S8

Supplementary Table S9

Supplementary Table S10

Supplementary Table S11
