# Supplementary material for: MicroRNA induction by copy number gain is associated with poor outcome in squamous cell carcinoma of the lung
Source: Sci Rep. 2018 Oct 18;8:15363. doi: 10.1038/s41598-018-33696-1 (PMC6194131; doi:10.1038/s41598-018-33696-1)
Supplement: Supplementary file 1 — Supplementary figures [file 41598_2018_33696_MOESM1_ESM.pdf]

MicroRNA induction by copy number gain is associated with poor outcome in squamous cell carcinoma of the lung

Endi Xia[a,1,2], Sotaro Kanematsu [a,3], Yusuke Suenaga[1], Asmaa Elzawahry[4], Hitomi Kondo[3], Noriko Otsuka[3], Yasumitsu Moriya[5], Toshihiko Iizasa[5], Mamoru Kato[4], Ichiro Yoshino[2] and Sana Yokoi*[,1,3]

[1] Cancer Genome Center, Chiba Cancer Center Research Institute, Chiba, Japan

[2] Department of General Thoracic Surgery, Graduate School of Medicine, Chiba University, Chiba, Japan

[3] Division of Genetic Diagnostics, Chiba Cancer Center, Chiba, Japan

[4] Department of Bioinformatics, National Cancer Center, Tokyo, Japan

[5] Division of Thoracic Diseases, Chiba Cancer Center, Chiba, Japan

[a] These authors contributed equally to this work.

Correspondence:

Sana Yokoi

Division of Translational Genomics, Chiba Cancer Center Research Institute, 666-2 Nitona-cho, Chuo-ku, Chiba Prefecture 260-8717, Japan

TEL: +81 43 264 5431

E-mail: syokoi@chiba-cc.jp

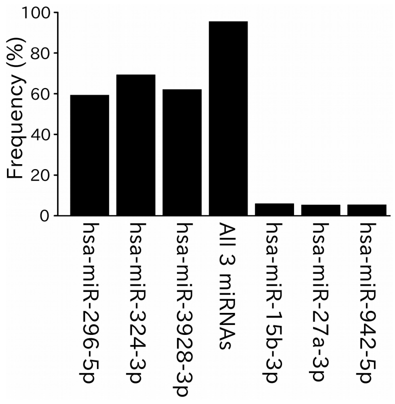

Supplementary Figure S1. Reproducibility of P-values in Cox proportional hazards models.

Barplot showing the percentage of significant P-values (<0.05) based on the log-rank test. The P-values of the three miRNAs associated with poor prognosis were reproducible, whereas those of randomly selected miRNAs (miR-15b-3p, miR-942-5p, and miR-27a-3p) were not reproducible.

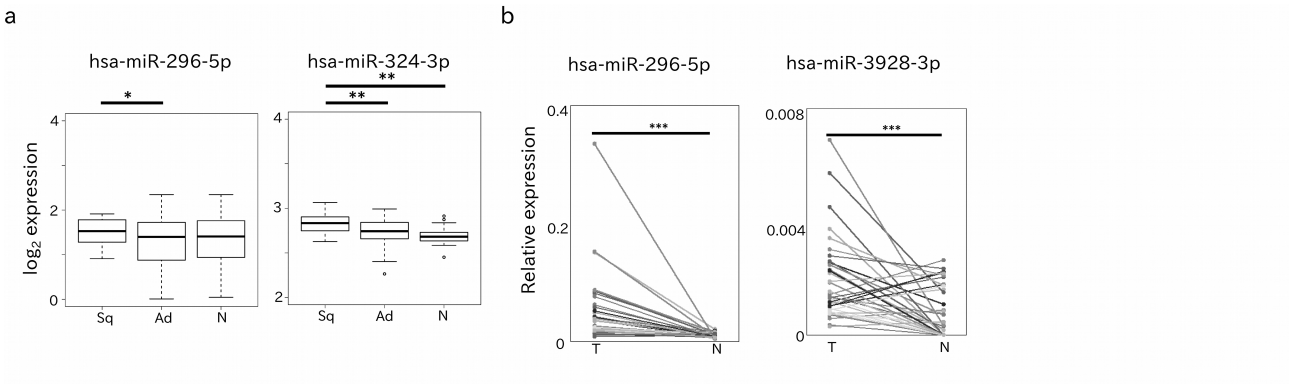

Supplementary Figure S2. Distribution of expression of onco-miRNAs in miRNA microarray data set and our own clinical samples.

(**a**) Boxplots showing higher expression of the three miRNAs of interest in Sq samples relative to those in Ad and normal tissues (N). P-values were calculated using the Wilcox-rank sum test: (*) P < 0.05; (**) P < 0.01. (**b**) Lines and dots showing the distribution of individual miRNA expression levels in 36 paired (tumor and normal) samples. P-values were calculated using the Wilcoxon signed-rank test: (***) P < 0.001.

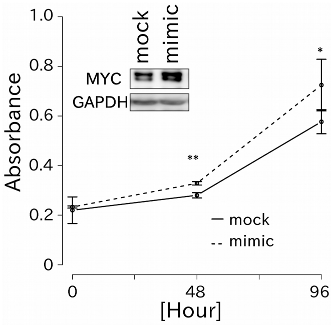

Supplementary Figure S3. Effect of miRNA mimic transfection on cell proliferation in ACC-LC73 cells. Polygonal line demonstrating that the combination of the three miRNA mimics promoted the proliferation in of ACC-LC-73 cells. P-values at each time point were calculated using the Wilcox-rank sum test: (**) $P < 0.01$, (*) $P < 0.05$. Western blot shows that the combination of the three miRNA mimics induced MYC protein expression (see also Supplementary Figure S8).

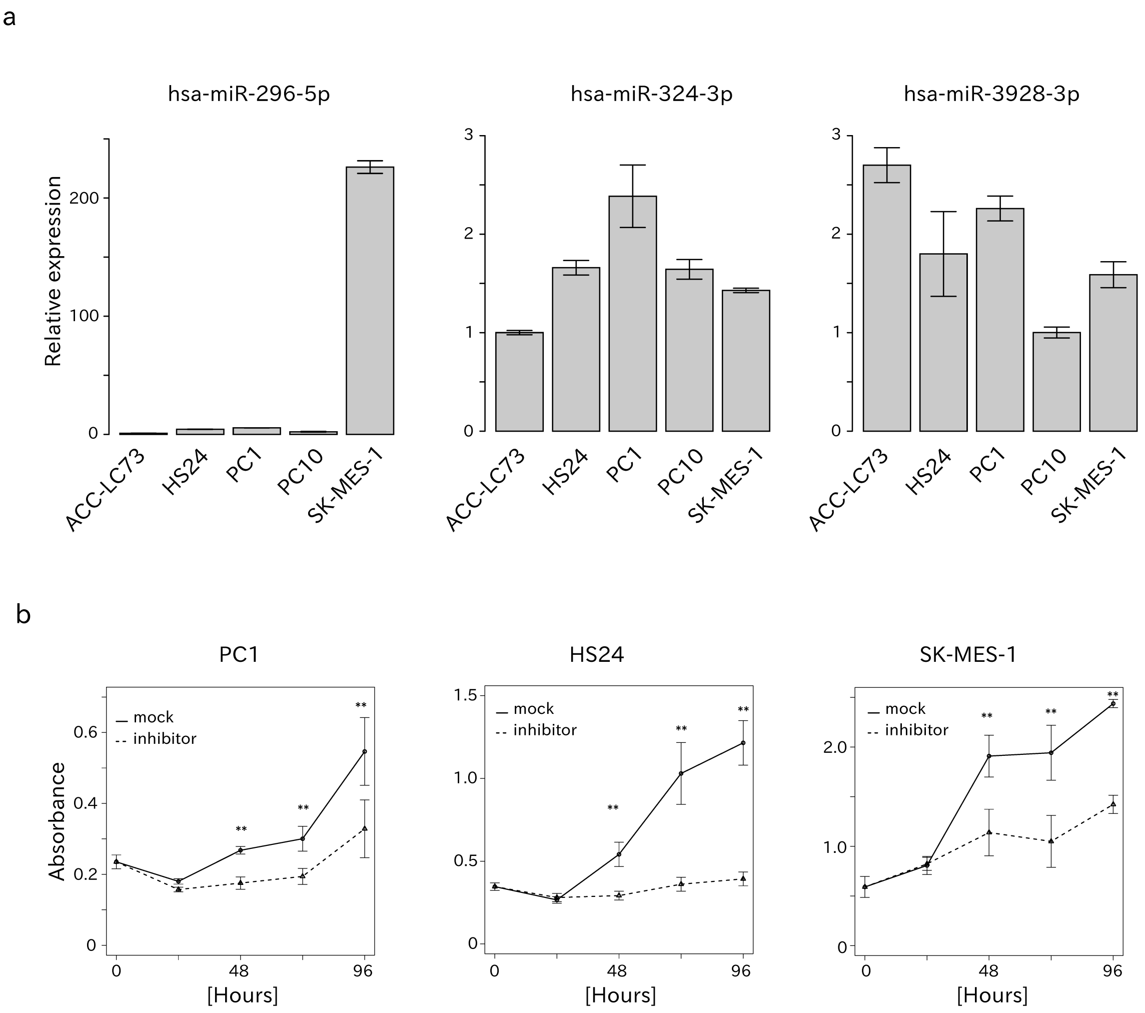

Supplementary Figure S4. Expression of each miRNA in multiple Sq cell lines, and the effect of miRNA inhibitor transfection on cell growth. (**a**) Barplot showing the expression levels of each miRNA in Sq cell lines. (**b**) Polygonal line demonstrating that the combination of the three miRNA inhibitors suppressed proliferation of PC1, HS24, and SK-MES1 cells. P-values at each time point were calculated using the Wilcox-rank sum test: (**) P < 0.01, (*) P < 0.05.

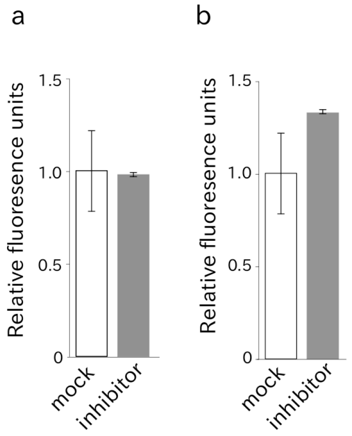

Supplementary Figure S5. Effect of miRNA inhibitor transfection on cell invasion and migration in PC10 cells. (**a**) Barplot showing that cell invasion was not changed by miRNA inhibitor transfection. (**b**) Barplot showing that cell migration was not changed by miRNA inhibitor transfection. Error bars show standard deviations.

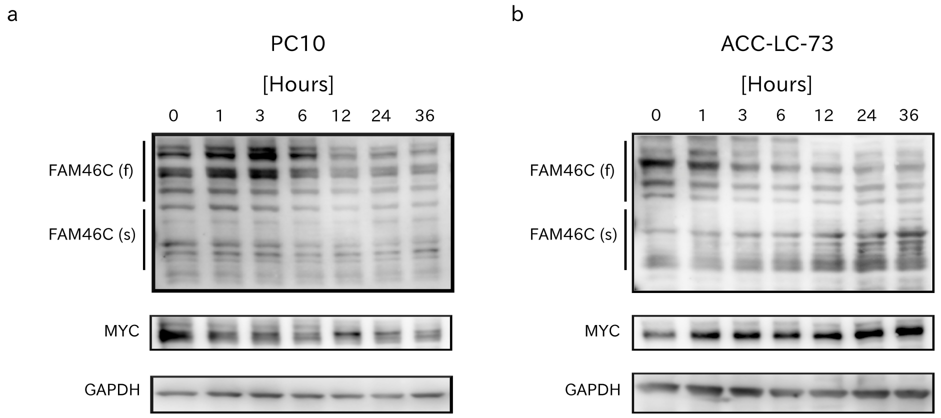

Supplementary Figure S6. Effect of miRNA inhibitor/mimic transfection on *FAM46C* and *MYC* protein expression. (**a**) Western blot showing that miRNA inhibitor induced FAM46C protein expression within a short time after transfection of PC10 cells. FAM46C protein expression levels were highest at 3 h after transfection, whereas MYC protein expression was suppressed. (**b**) Western blot showing that the miRNA mimic suppressed FAM46C protein expression within a short time after transfection of ACC-LC-73 cells. FAM46C protein expression levels was were highest at 3 h after transfection, whereas MYC protein expression was induced.

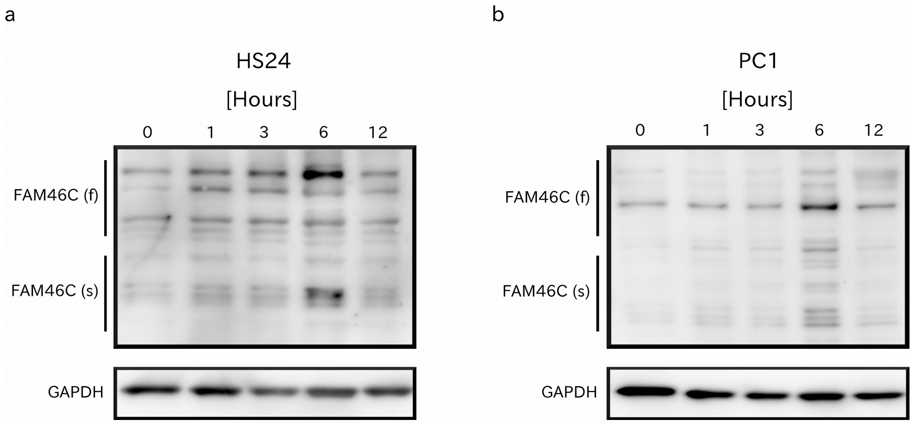

Supplementary Figure S7. Validation of the effect of miRNA inhibitor transfection on *FAM46C*. (**a**) Western blot showing that miRNA inhibitor induced FAM46C protein expression within a short time after transfection of HS24 cells. FAM46C protein expression levels were highest at 6 h after transfection. (**b**) Western blot showing that miRNA inhibitor induced FAM46C protein expression within a short time after transfection of PC1 cells. FAM46C protein expression levels was were highest at 6 h after transfection.

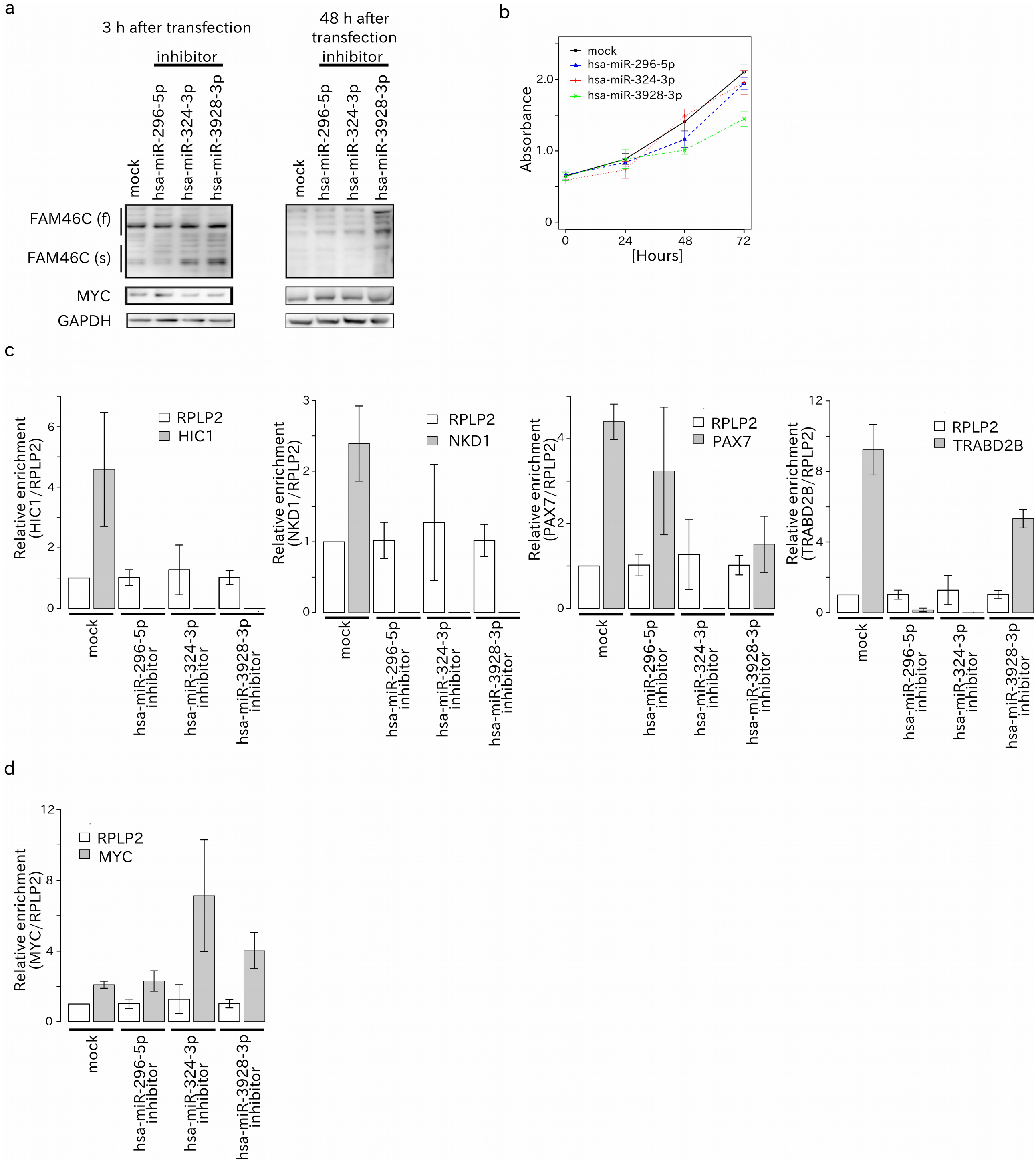
a
3 h after transfection
inhibitor
mock
hsa-miR-296-5p
hsa-miR-324-3p
hsa-miR-3928-3p
48 h after transfection
inhibitor
FAM46C (f)
FAM46C (s)
MYC
GAPDH
b
mock
hsa-miR-296-5p
hsa-miR-324-3p
hsa-miR-3928-3p
Absorbance
[Hours]
c
RPLP2
HIC1
Relative enrichment (HIC1/RPLP2)
NKD1
Relative enrichment (NKD1/RPLP2)
PAX7
Relative enrichment (PAX7/RPLP2)
TRABD2B
Relative enrichment (TRABD2B/RPLP2)
hsa-miR-296-5p inhibitor
hsa-miR-324-3p inhibitor
hsa-miR-3928-3p inhibitor
d
RPLP2
MYC
Relative enrichment (MYC/RPLP2)

Supplementary Figure S8. Effect of each miRNA on expression of *FAM46C*. (**a**) Western blots showed that each miRNA inhibitor increased FAM46C protein expression at 3 h (*left*) and 48 h after transfection (*right*) (**b**) Polygonal line demonstrating that the effect of the suppression of cell proliferation of PC10 cells. (**c**) Barplot showing the ability of potential target to bind to AGO2 via miRNA. (**d**) Barplot showing the ability of *MYC* mRNA to bind to AGO2 via miRNA.

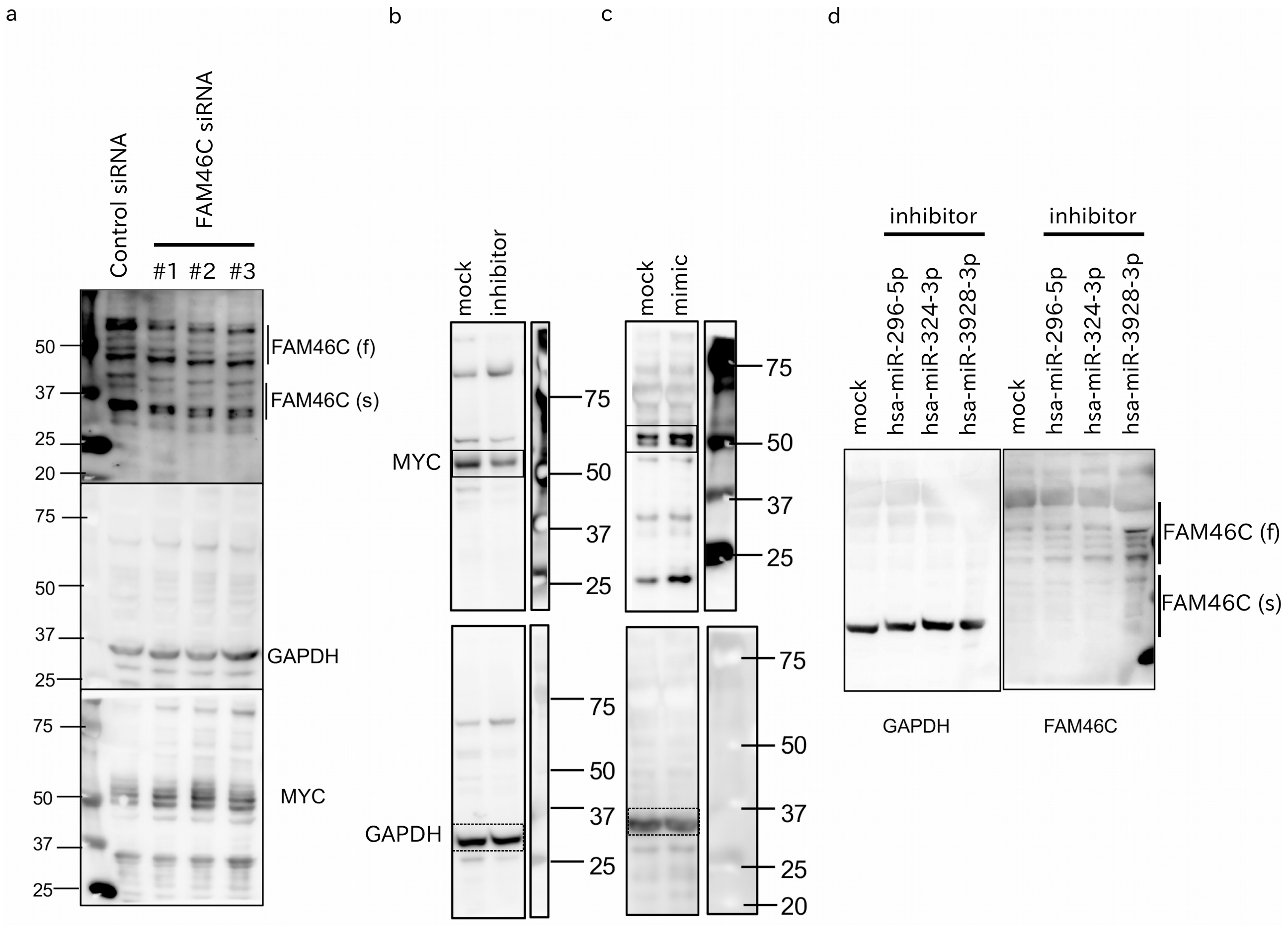

Supplementary Figure S9. Full blot figures. Full scanned gels of western blots shown in Figures 4 (**a,b**), S3 (**d**), and S4 (**c**). Some gel images were cropped to merge the MW markers with the protein bands while removing samples unrelated to the study, which were run on the same gel. Black frames indicate cropped parts used in the original figures. FAM46C encodes full-length (f) and short (s) proteins (reference 11). The multiple bands indicate that FAM46C protein was modified by ubiquitylation or phosphorylation, as these post-translational modifications are reported in public databases (nextprot, NX_Q5VWP2; PhosphoSitePlus, Q5VWP2).
